# Correlates of Blood Pressure and Cholesterol Level Testing Among a Socially-Disadvantaged Population in Poland

**DOI:** 10.3390/ijerph17062123

**Published:** 2020-03-23

**Authors:** Małgorzata Znyk, Kinga Polańska, Leokadia Bąk-Romaniszyn, Dorota Kaleta

**Affiliations:** 1 Department of Hygiene and Epidemiology, Medical University of Lodz, 90-647 Lodz, Poland; kinga.polanska@umed.lodz.pl (K.P.); dkaleta@op.pl (D.K.); 2 Department of Nutrition in Digestive Tract Diseases, Medical University of Lodz, 93-338 Lodz, Poland; leokadia.bak-romaniszyn@umed.lodz.pl

**Keywords:** blood pressure testing, cholesterol level testing, lifestyle factors, healthy lifestyle index, correlates

## Abstract

As part of cardiovascular disease prevention, the performance of BMI determination, blood pressure measurement, biochemical tests, as well as a lifestyle-related risk assessment are recommended. The aim of this study was to evaluate the correlates of blood pressure and cholesterol level testing among a socially-disadvantaged population in Poland. This cross-sectional study was performed between 2015 and 2016 among 1710 beneficiaries of government welfare assistance. Face-to-face interviews conducted by trained staff at each participant’s place of residence allowed for completion of questionnaires that covered socio-demographic, health and lifestyle-related information. Sixty-five percent of the participants declared a blood pressure and 27% of them cholesterol level testing at least once within the year proceeding the study. A higher chance of having blood pressure testing was observed among the women (OR = 1.5; p = 0.002) and people with high blood pressure (OR = 3.9; p < 0.001). The women (OR = 1.4; p = 0.04) and older people (OR = 1.9; p = 0.02; OR = 2.6; p < 0.001, OR = 2.7; p = 0.002, for the following age groups: 30-39, 40-49, 50-59 years respectively), the respondents who declared health problems such as heart attack (OR = 3.0; p = 0.04), high blood pressure (OR = 2.3; p < 0.001) and type 2 diabetes (OR = 3.3; p = 0.004) and those with a family history of chronic diseases (OR = 1.5; p = 0.03) had a higher chance of cholesterol level checking. Higher healthy lifestyle index, indicating that the study participants have followed almost all of the studied lifestyle-related recommendations, was a significant correlate of cholesterol level testing (OR = 1.7; p = 0.006). Actions that promote lifestyle changes, blood pressure, and cholesterol level testing should take into account the needs of the disadvantaged population and should especially target men, people with existing chronic diseases, and those with unfavorable lifestyle characteristics. With respect to the socially-disadvantaged population, the social assistance institutions and outpatient clinics are the best places to conduct activities promoting a healthy lifestyle. The most commonly applied strategies to promote lifestyle changes can cover risk assessment, increasing awareness, emotional support and encouragement, as well as a referral to specialists.

## 1. Introduction

Chronic diseases constitute a serious health problem in the world. According to the World Health Organization (WHO), around 41 million deaths that occurred in 2016 were due to non-communicable diseases (NCD), accounting for 71% of the total 57 million deaths [1,2]. Cardiovascular diseases (CVD) are one of the main causes of morbidity and mortality, which account for 44% of all deaths from NCDs [1,3]. CVDs kill over 4 million people in Europe each year. Generally, the number of deaths due to CVSs is higher among women (2.2 million) than among men (1.8 million), although deaths due to cardiovascular causes in people younger than 65 years of age are more common among men than women (490,000 vs. 193,000) [4].

Arterial hypertension (AH) is the key dominant risk factor for CVDs [5]. The diseases attributed to hypertension include: stroke, heart failure, ischemic heart disease, hypertensive retinopathy, renal failure [5]. AH is also associated with diabetic, endocrine, nephrological diseases and osteoporosis [6]. The American Heart Association/American College of Cardiology (AHA/ACC) guidelines treat all blood pressure (BP) values above 130/80 mmHg as hypertension [7]. Two stages of hypertension have been defined: stage 1 of hypertension in the range of 130–139/80–89 mmHg, and stage 2 for any pressure equal or above 140/90 mmHg. According to the guidelines of the European Society of Cardiology (ESC) and the European Society of Hypertension (ESH) from 2018 and the Polish Society of Hypertension (PTNT) from 2015, normal BP values in an adult were defined as less than 140/90 mmHg [8]. It is recommended to divide BP into optimal (below 120/80 mmHg), normal (120–129/80–84 mmHg), high normal (130–139/85–89 mmHg) and grades of hypertension 1–3 [8]. Values in the 140–159/90–99 mmHg ranges are defined as first stage hypertension, in the 160-179/100-109 mmHg ranges as second stage hypertension, and above 180/110 mmHg, third stage [9]. More precisely, following ESC guidelines, people under 65 years of age should have systolic blood pressure (SBP) of 120–129 mmHg, regardless of whether they have only arterial pressure or are associated with co-morbidities. Target SBP values within 130–139 mmHg are recommended for older patients over 65–80 years of age. Target SBP values in the range 130–139 mmHg are recommended for patients over 80 years of age subject to good tolerability. Target diastolic blood pressure (DBP) values below 80 mmHg should be considered in all hypertensive subjects, regardless of the risk level and co-morbidities [8]. It is estimated that AH equal to or exceeding 140/90 mmHg occurs in more than 25% of the world’s population. About 10 million people in Poland (32% of the adult population) suffer from that condition [10]. In order to asses a cardiovascular risk of patients with hypertension the following have been taken into account: uric acid levels, accelerated heart rate as well as socioeconomic and psycho-sociological factors [8].

An important factor, contributing to about one third of deaths caused by all CVDs, is elevated blood cholesterol [11]. Cholesterol may contribute to the development of pathological conditions resulting from both: accumulation of its particles in the body as well as disorders in their metabolism. Its participation in pathogenesis has been described, among others in the case of neurodegenerative diseases, CVDs, kidney diseases and cancer [12]. The basic tests recommended in the diagnosis of lipid disorders include: total cholesterol (TC), triglycerides (TG), HDL cholesterol (HDL-C), LDL cholesterol (LDL-C), and non-HDL cholesterol (non-HDL-C). LDL-C is the main lipid parameter used in the diagnosis of lipid disorders, risk assessment and treatment [3,13]. The recommended level of TC in the blood is below 200 mg/dL (5.2 mmol/L). Elevated cholesterol is defined as 200–250 mg/dL (5.1–6.5 mmol/L). We talk about a significantly elevated level when its value exceeds 250 mg/dL (> 6.5 mmol/L). The ESH/ESC guidelines state that cholesterol intake should be reduced to <300 mg/day, especially in people with high plasma levels [3]. The Polish Diabetes Association also reports that, in patients with elevated LDL cholesterol (≥ 100 mg/dL), dietary cholesterol should be reduced to <200 mg/day [14].

Due to ageing of the population, a non-healthy lifestyle and the increase in average body weight, the frequency of diagnosis of AH in the world will increase. Forecasts indicate that by 2025 the number of people with hypertension will have increased by 15–20%, reaching about 1.5 billion [15].

A lifestyle modification is the most effective method for preventing development of AH, while in the early diagnosis of the disease screening BP measurements performed at least once a year is of key importance [16].

BP measurement at a physician’s office or outpatient clinic remains the gold standard for diagnosing AH. Current ESH/ESC guidelines for the management of hypertension point to the high value of extra-office BP measurements—home blood pressure monitoring (HBPM) and 24-h ambulatory blood pressure monitoring (ABPM) in establishing the diagnosis and indications for AH treatment [17].

As part of the Cardiovascular Disease Prevention Program introduced to primary healthcare, biochemical tests (concentration of cholesterol, triglycerides, and glucose), BP measurement, Body Mass Index (BMI) determination, and risk assessment according to the Systematic COronary Risk Evaluation system (SCORE), are recommended. Persons covered by the program undergo educational activities in the field of a healthy lifestyle and patients diagnosed with CVDs are referred for a further diagnosis and specialist treatment [18].

Physicians should not only focus on the clinical treatment of an existing disease, but also recognize the health needs of their patients through an active discussion, identifying positive health behaviors, monitoring health state of their patients and providing support offering lifestyle advice [19,20,21,22]. A properly educated patient will participate in the process of prevention and treatment more effectively. Primary care physicians know their patients and can play a key role in counseling a healthy lifestyle. In Polish regulations in the field of activities aimed at the prevention of diseases, it is a primary care physician who identifies health hazards and patients’ risk factors and also implements actions aimed at their reduction [23].

Cooperation between medical staff and a patient and compliance with a physician’s recommendations is a significant challenge in the treatment of chronic diseases. The aim of the study was to evaluate the correlates of BP and cholesterol level testing among a socially-disadvantaged population in Poland. This could be crucial for implementation of targeted preventive measures among this vulnerable population.

## 2. Material and Methods

### 2.1. Study Design and Population

Characteristics of the examined region, methodology, and the study sample have been published elsewhere [24,25,26]. Briefly, this cross-sectional study that covered beneficiaries of government welfare assistance was performed between October 2015 and February 2016 among residents of Piotrkowski district aged 18–59 years. For the selection of the study sample, the poverty threshold as adopted by the social assistance institutions was applied (income of no more than 634 PLN (148 Euro) for a single person monthly, and 514 PLN (120 Euro) for a family member monthly). Taking into account age and income limit, 3636 individuals met the inclusion criteria and 1817 agreed to participate in the study (50.0%). Of this group, 1710 people (94.1%) had core data available for the current analyses.

The Bioethics Committee of the Medical University in Lodz granted approval for this study (RNN/243/15/KE) and informed consents were obtained from all the study participants.

### 2.2. Study Variables

Face-to-face interviews conducted by trained staff at each participant’s place of residence allowed for completion of the questionnaires. The questionnaire covered socio-demographic, health status and lifestyle-related information. In the analyses under this study, a respondent’s sex (male, female) and age (categorized as: <30, 30–39, 40–49, 50–59 years) were selected. The second group of questions focused on a health status including: subjective health state (categorized as: fair/rather fair, neither fair nor poor, rather poor/poor) and a number of specified health problems noted within the past month (categorized as: none, 1–3, 4–6, >7). The respondents were also asked if they have had heart attack, high blood pressure (HBP), or type 2 diabetes (yes, no). The family history of chronic diseases, including: heart attack, coronary artery disease, stroke, cancer, HBP, diabetes, high cholesterol level, and overweight/obesity was also reported (categorized as: yes–if any and no–if neither was indicated). Two dependent variables were considered in the current analyses: (1) BP and (2) cholesterol level testing. An answer stating that the respondents had BP or cholesterol level testing done within the year proceeding the study was considered as positive and the other options (never, between one and five years, more than five years ago, I don’t know) as negative.

The participants were also asked several questions that allowed us to describe five lifestyle-related characteristics: (1) smoking status, (2) diet, (3) height and weight, (4) alcohol consumption, and (5) recreational physical activity. The pattern of each lifestyle-related characteristics was defined as a single healthy lifestyle indicator (HLI) coded as 1 if a respondent followed healthy lifestyle recommendations and as 0 if the recommendations were not followed. Finally, the healthy lifestyle index (range, 0–5) was created by adding up all the five HLIs.

The study participants were asked about their smoking status. Those who answered that they had never smoked cigarettes received 1 for HLI. For the current smoking status (including daily or occasional smoking) the following coding was given: yes: HLI = 0; no: HLI = 1 [24]. Details regarding each participant’s diet and its categorization according to the existing guidelines were published previously [25]. For the purpose of the current analysis HLI equal 1 was given to the participants with healthy or average dietary habits and HLI equal 0 to those who had not followed the recommendations. Healthy weight was defined as BMI (based on a participant’s height (m) and weight (kg)) between 18.5 and 24.9 (kg/m^2^) (HLI = 1) [27]. For alcohol consumption, information regarding frequency, intensity (units of alcohol per day and binge drinking), and type of alcohol (wine, beer, spirit) was collected. According to the existing guidelines, frequent and high-quantity alcohol consumption is related to a poorer health-related quality of life. It is also recommended that women should consume up to one and men up to two units of alcohol per day [28]. HLI equal to 1 was dedicated to the participants who followed the recommendations. Finally, the participants were asked several questions about their leisure-time physical activity (LTPA) (including type, duration, and frequency of LTPA) and commuting physical activity (CPA) as described previously [26]. The participants who achieved criteria for the recommended level of recreational physical activity based on LTPA or CPA received, HLI = 1 [29].

### 2.3. Statistical Analysis

For the variables included in the study, the percentage share of individual categories of the responses in the study group was calculated.

The unadjusted and adjusted odds ratios (OR) and 95% confidence intervals (95% CI) were calculated to identify the correlates of performing BP testing and cholesterol level testing within the year proceeding the study. The Mantel–Haenszel chi-square statistics were used. Multivariate models included all of the studied variables. The significance level of statistical inference was set at p<0.05. STATISTICA version 10.0 (Dell Software, Arizona, CA, USA) was used to perform the statistical analysis.

## 3. Results

### 3.1. Characteristics of the Study Population

The women constituted 67% of the study population (Table 1). The mean age of the respondents was 39.2 ± 7.7 years. Most of the subjects described their health status as fair or rather fair and one in tenth as rather poor or poor. About 1% of the participants had heart attack, 2.5% type 2 diabetes and 12%were diagnosed with HBP. Family history of any chronic diseases was indicated by almost 70% of the participants. More than 37% of the beneficiaries of government welfare assistance were smokers. The health lifestyle recommendations were followed by 56% for alcohol consumption, 26% for a recreational physical activity and 9% for a diet. The recommended range of BMI (between 18.5 and 24.9 (kg/m^2^)) was reported by 43% of the participants. The healthy lifestyle index (summed up 5 analyzed lifestyle features) between 0 to 3 was noted for 84% of the subjects and only 10% gained 4 out of 5 points. The maximum number of points (which means that the respondents followed all 5 lifestyle-related recommendations) was achieved by 11 people only (0.6%).

### 3.2. Correlates of Blood Pressure and Cholesterol Level Testing

Sixty-five percent of the beneficiaries of government welfare assistance declared BP testing at least once within the year proceeding the study (Table 1). Much fewer participants had their cholesterol level checked (27%).

The results of the univariate and multivariate analyses of the correlates of BP and cholesterol level testing among the socially-disadvantaged population in Poland are presented in Table 2. A higher chance of having BP testing was observed for the women (OR = 1.5; p = 0.002) and people with a diagnosed HBP (OR = 3.9; p < 0.001). The individuals with the family history of chronic diseases had BP checked more frequently; however, the results were of borderline significance (OR = 1.3; p = 0.06). More and stronger correlates were noted for cholesterol level testing. The women (OR = 1.4; p = 0.04) and older people (OR=1.9; p=0.02; OR = 2.6; p < 0.001, OR = 2.7; p = 0.002, for the following age groups: 30-39, 40-49, 50-59 years, respectively) had a higher chance of having cholesterol level testing as compared to the men and people younger than 30 years of age. The respondents who declared health problems such as heart attack (OR = 3.0; p = 0.04), HBP (OR = 2.3; p < 0.001), type 2 diabetes (OR = 3.3; p = 0.004), and those with family history of chronic diseases (OR = 1.5; p = 0.03) also had a higher chance of cholesterol level checking. A higher healthy lifestyle index, indicating that the study participants have followed almost all of the studied recommendations related to the lifestyle, was a significant correlate of cholesterol level testing (OR = 1.7; p = 0.006).

## 4. Discussion

This study provides an analysis of the correlates of BP and cholesterol level testing among a socially-disadvantaged population in Poland. Primary care physicians are often the only line of contact between a patient and healthcare system in the prevention of chronic diseases, including CVDs. They should play a key role in lifestyle examination and modification as well as the early identification and monitoring of health problems. In the current study the following correlates of a higher chance of BP and cholesterol level testing were identified: female gender, older age as well as health problems such as: heart attack, HBP and type 2 diabetes and a family history of chronic diseases. What is more, a higher healthy lifestyle index, indicating that the study participants have followed almost all of the studied recommendations related to the lifestyle, was a significant correlate of cholesterol level testing. This information is crucial for implementation of targeted preventive measures among this vulnerable population.

Epidemiological studies conducted in Poland in 1997–2017 have shown that hypertension occurs in 29% (NATPOL PLUS, 2002) to 45% (WOBASZ II, 2013–2014) of the adult population and even in 75% of people aged 65 years and over (PolSenior, 2007–2011) [9]. The NATPOL 2011 project has shown that prevalence of hypertension in Poland in adults below 80 years old was 32% [30].

In the WOBASZ II survey, hypercholesterolemia has been found in 70% of men and 64% of women. Isolated hypertriglyceridemia has been diagnosed in 6% of men and 2% of women. The prevalence of hypercholesterolemia has not changed significantly from 2003–2005. However, an increase in the incidence of hypertriglyceridemia has been found in men, and an increase in the incidence of low HDL-C levels has been observed in both sexes [31].

In the study of Kearney et al. performed by a representative method in 40 countries, it has been estimated that the incidence of hypertension in the world in 2000 was 26% (972 million people), and in 2025 it will increase to 29% (1560 million people) [15]. According to the WHO experts, increased BP is responsible for 13% of all deaths in the world [15,30]. Collins and MacMahon have shown that effective treatment of hypertension and a reduction in diastolic pressure of 5–6 mmHg reduces the risk of complications of coronary heart disease by 16%, and stroke by 38% [30]. A meta-analysis of 123 randomized control studies has shown that every 10 mmHg reduction in SBP resulted in a 20% reduction in the risk of serious cardiovascular incidents, reduction in the incidence of coronary heart disease, reduction in the frequency of strokes and heart failure, which ultimately led to up to 13% reduction in all-cause mortality [32].

In our study, 12% of the subjects declared HBP, which is lower than in other studies and can result from self-report. What is more, considering their financial situation, the study participants can pay more attention to their everyday needs than to their health state. Sixty-five percent of the beneficiaries of government welfare assistance declared having a BP test performed at least once within the year proceeding the study. About 87% of the people who declared having AH indicated that they had previously had BP tests done. BP control up to <140/90 mmHg is achieved in less than a quarter of the people diagnosed with hypertension and in a third of those treated for hypertension, even in countries with a well-structured healthcare system [33,34,35]. In Brazil, rates of effective BP control from 10% to 57% have been reported [36]. A study by Martínez-Valverde et al. reports effectiveness of a continuous intervention in medical education (CME) to improve adequate care for hypertension in controlling BP in hypertensive patients in primary care clinics in Mexico. Treatment by a family physician who participated in CME intervention reduced the likelihood of a lack of BP control by 53%. Adopting dietary recommendations reduced the likelihood of uncontrolled BP by 57% [37].

Ways to maintain the recommended cholesterol level and prevent or control HBP are simple and include: maintaining a regular physical activity, healthy weight, not drinking alcohol, and not smoking.

In our study, the healthy lifestyle index between 0 to 3 was noted for 84% of the respondents, and only 10% scored four out of five points. Only 0.6% of the people followed all 5 lifestyle recommendations. Those individuals who followed 4–5 recommendations performed BP testing and cholesterol level testing more often as compared to those who have not followed any of the recommendations (71.2% vs. 64.9%; p = 0.3 and 38.6% vs. 24.7%; p < 0.05). This means that they generally represented positive attitudes towards health. On the other hand, people with an unhealthy lifestyle should constitute a target group for BP and cholesterol level checking as they have a higher risk of it and other related diseases. The testing can be also recognized as an element of healthy lifestyle counseling (as the elevated BP and/or cholesterol level can stimulate people to modify their lifestyles).

The recommended lifestyle changes that are effective in lowering BP include: reducing salt and alcohol intake, high fruit and vegetable intake, weight reduction and maintaining recommended body weight as well as a regular physical activity [38]. Also, smoking has a sharp, prolonged BP elevating effect, which may cause an increase in pressure during the 24-h monitoring period. Smoking cessation and other lifestyle changes have also other beneficial effects apart from lowering BP (e.g., reducing the risk of CVD or cancer) [5]. Lifestyle recommendations that effectively improve the plasma lipid profile include: reducing trans and saturated fat intake, increasing vegetable and fruit and fiber intake, reducing excess weight, and increasing physical activity. Smoking cessation has a positive effect on lipid parameters, including HDL-C concentration. [3].

Shah et al. have shown that a light exercise (such as aerobics) reduces BP and heart rate [39]. A regular low intensity and short duration physical activity reduces BP to a smaller extent than a moderate to intense physical activity but is associated with at least a 15% reduction in mortality in cohort studies [40,41]. Hurley et al. have proved that exercises act as prevention and treatment of metabolic syndrome, insulin resistance, abdominal obesity, hyperlipidemia, rheumatoid arthritis, and hypertension. Much smaller use of this method can be seen in the treatment of lipid disorders (however, it is a method that supports triglyceride control) [42]. Patients with hypertension should be advised to have at least 30 min of a moderate dynamic aerobic exercise (walking, cycling, running, or swimming) for 5–7 days a week.

In our analysis only 26% of the participants followed the recommendations for LTPA. An in-depth analysis focused on a LTPA and its correlates in our study population has been published previously [26]. This indicates that the men had a higher risk of inactivity during LTPA compared to the women. Higher odds of commuting physical inactivity (CPIA) were associated with unemployment, moderate and heavy drinking and having several health problems.

A diet, as a modifiable factor ensuring good health maintenance, reduces the risk of non-communicable chronic diseases and prevents a premature death [25]. Eating habits affect cardiovascular risk by affecting serum LDL and HDL cholesterol, BP, body weight, and glycemic control in diabetes. The Mediterranean diet has been shown to prevent development of CVD, breast cancer, colorectal cancer, depression, obesity, diabetes, asthma, erectile dysfunction and cognitive impairment [43,44]. The Mediterranean diet significantly reduces BP in 24-h measurements, glucose, and lipids [45]. Low consumption of potassium, magnesium, calcium, and at the same time, high consumption of sodium, saturated and trans-fatty acids, and cholesterol causes an increase in BP, while the DASH (dietary approaches to stop hypertension) diet lowers it [10].

A proper diet, rich in vegetables and fruit (especially containing potassium), with a reduced amount of saturated fat, helps to regulate BP. Studies show that men with low incomes and low levels of education eat vegetables and fruit less frequently [46,47], and less often follow fish guidelines [48].

A recent meta-analysis of the available studies has shown that a reduction in sodium intake of ~ 1.75 g per day (4.4 g salt per day) is associated with a reduction of SBP/ DBP by an average of 4.2/ 2.1 mmHg and even greater hypotensive effect (−5.4/−2.8 mmHg) observed among people with hypertension [49]. An adequate diet should be accompanied by other changes, such as a physical activity and weight loss, drinking green or black tea may also have a small but significant BP-lowering effect [5,50,51]. In our study population only 9% of the participants met the criteria for a healthy diet. An in-depth analysis of the correlates of a diet in our study population has been already published [25]. Unhealthy eating habits dominated in all the groups regardless of the level of education of the subjects (p < 0.001). In the study by Michoty-Kotulska et al., irregularities have been also found in the nutritional behavior of physicians, such as: excessive consumption of sweets, wheat bread, sausages, and insufficient fruit, vegetables, fish, dairy, and wholemeal cereal products, which may lead to the development of diet-related diseases [52].

The most important clinical complications of obesity, in addition to hypertension, include: lipid disorders, insulin resistance, inflammation, and blood clotting, albuminuria, as well as development of diabetes and the occurrence of cardiovascular events [43]. In the meta-analyses, the average reduction of SBP and DBP with an average weight reduction of 5.1 kg was 4.4 and 3.6 mmHg, respectively [53]. The results of epidemiological studies indicate that obese people suffer from hypertension more often than people with normal body weight [54]. In our study, the recommended BMI range (18.5 and 24.9 (kg/ m2)) was reported by 43% of the participants. According to WHO data, the percentage of overweight men in Poland is 43%, and women - 31%; percentage of obese people 25% and 26% respectively [55]. In Poland, in the NATPOL 2011 study, among people with BMI <25kg/ m^2^, overweight, and obesity, the percentage of patients with hypertension (NT) was: 11%, 40%, and 60% in men and 18%, 38%, and 61% in women, In the adult population of Poles, significant relationships between SBP and DBP and BMI have been found at all ages, both in younger and older people [30]. Obesity increases the risk of developing type 2 diabetes 3–7 times, and a person with a BMI > 35 has a 20-fold higher risk of developing diabetes than a person with a BMI within the normal range, while weight reduction is the main component of type 2 diabetes [54]. The survey of Abramczyk has shown that persons with normal body mass showed BP, triglyceride concentrations and total cholesterol concentrations close to normal more frequently. Obesity was most frequently recognized in patients who declared no physical activity [56]. Even a small reduction in body weight (by 5–10% of the initial value), reduces lipid disorders and has a positive effect on other cardiovascular risk factors common in people with lipid disorders [3]. Hypertension is also a big problem in diabetes, causing a significant threat to the cardiovascular system [57]. Hypertension increases the macro- and microvascular complications of diabetes, such as neuropathy, nephropathy, coronary artery disease, stroke and retinopathy [6]. The basis for CVD prevention in diabetic people, in addition to glycemic control, are the same general principles as for people without diabetes. Good control of BP and lipid levels is particularly important [43,58].

It is believed that reducing alcohol consumption and smoking cessation reduce BP by several mmHg [10]. The average prevalence of smoking in Europe is still high, at 20–35% [59]. Studies using ABPM have shown that smokers with normal BP as well as untreated hypertension have higher BP values during the day compared to non-smokers [60]. Smoking cessation has a positive effect on cardiovascular risk, and on HDL-C concentration, but special attention should be paid to preventing weight gain in those who quit smoking [61]. In our study, over 37% of the beneficiaries of government welfare assistance were smokers [24]. In the analysis by Milcarz et al. (on our study population), awareness of the health risk related to smoking was declared by 92% of the study participants, including 94% of women and 90% of men. More than a third of smokers have indicated that they wanted to quit. About a quarter of respondents did not make any decision to quit smoking. Our previous analyses and the results from other studies stressed an urgent need to broaden knowledge about health risks of active and passive smoking, and that additional efforts should be made by healthcare professionals to disseminate that information together with effective help in quitting smoking (including counseling and treatment) [24,62,63].

Studies have shown that alcohol intake affects various biomarkers (lipids, BP, homocysteine, diabetes, haemostatic factors) associated with the risk of coronary heart disease. Reducing alcohol consumption, even in people who drink low to moderate amounts of alcohol, can have a positive effect on CVDs [64]. The WOBASZ (2003–2005) study found that moderate drinkers had a 37% higher risk of hypertension, a 25% higher risk of elevated triglyceride level, a 40% lower risk of low HDL-C levels, and a 35% lower risk of diabetes compared to light drinkers. Heavy alcohol consumption increased the likelihood of hypertension by 52%, hyperhomocysteinemia by 95%, elevated triglycerides by 46%, and decreased the likelihood of low HDL-C by 44% [65].

In our study, 56% of the participants followed the recommendations for alcohol consumption. Drinking men with hypertension should be advised to limit their alcohol consumption to 14 units per week, and women to 8 units per week (1 unit corresponds to 125 mL of wine or 250 mL of beer) [5]. Moderate alcohol consumption up to 20 g/d in men and 10 g/d in women is acceptable for people who consume alcoholic beverages, provided that the plasma TG concentration is not increased [3].

The education of patients suffering from hypertension and hypercholesterolemia should be multifaceted, including, in addition to raising awareness of the essence of the disease and the risk of its complications, also elements of a healthy lifestyle, including a physical activity, diet and smoking [16,66]. Aspects related to the preparation and proper techniques of measuring BP and factors affecting the value of the measurement are also important. The studies of Połetek et al. have shown that the largest percentage of patients derive knowledge about the correct measurement of BP from medical staff, mainly physicians, at 80% [67].

Several interventions at the population level have been effective in influencing individual lifestyles. Awareness and knowledge on how lifestyle risk factors lead to CVDs have increased in recent decades, and have contributed to reducing the prevalence of smoking and decreasing cholesterol levels. In addition, legal solutions favoring a healthy lifestyle, such as limiting salt intake and smoking bans, are cost-effective ways to prevent CVDs [68,69,70,71].

There are many perceived barriers to follow healthy lifestyle recommendations in vulnerable populations (such as beneficiaries of government welfare assistance), including individual and lifestyle issues, social and community barriers, living and working conditions, as well as cultural, socio-economic and environmental factors. What is more, they can believe that their health (and lifestyle-related behavior) is their personal responsibility. They can also experience lack of necessity to seek advice/counseling/treatment from professionals, lack of awareness of the available control measures/treatment, or misperceptions about the costs, safety, and side effects of the offered help/medications/procedures.

Patients should undergo regular follow-up visits (at least once a year) to measure BP in the office and outside it, as well as to check the CVDs risk profile [7]. The psychological aspect is also important. Measurements at a physician’s office give a patient the feeling that antihypertensive therapy is being properly controlled [72]. The latest recommendations also strengthen the role of measurements outside a physician’s office, i.e., measurements taken by a patient himself, as well as ABPM. These methods are very useful for verifying whether a patient has white coat hypertension or suspected masked hypertension. During a visit, a patient should receive recommendations for measuring BP and cholesterol level testing as basic method in the prevention of CVDs and atherosclerosis. Recommendations for lifestyle changes, which in the case of many patients will be a sufficient treatment, are also important [73].

The strengths and limitations of the study need to be pointed out. Firstly, this study has covered a disadvantaged population, which is usually less frequently covered by research and preventive measures. On the other hand, individuals belonging to such a population have a worse state of health and a worse lifestyle than people from the general population and therefore, should be included in preventive measures and pro-health activities to an ever-larger extent. The response rate achieved in this study (50%) is comparable to the one observed in other epidemiological studies. The limitations of the study are mostly related to the cross-sectional study design, which limits the conclusions for causality; and self-reported data both for lifestyle related information and health status, which can be related to the response bias. Moreover, considering the extent of the study (that covered a number of actions, including epidemiological studies aimed at understanding the health status, health behaviors of the local community, as well as a broad number of activities in the field of health promotion) some data were not available for the current assessment. As an example, in addition to BMI, the waist/hip circumference measurement (which was not taken under the current study) would be more accurate to classify the people as overweight/obese.

## 5. Conclusions

Preventive measures seem to be the easiest way to reduce the risk of developing chronic diseases as well as to improve effectiveness of the treatment of the disease. Primary education and prevention focused on a lifestyle modification, as well as the improved detection of hypertension and hypercholesterolemia should be a priority in improving the population health.

In the current study, the following correlates of a higher chance of BP and cholesterol level testing were identified: female gender, older age, as well as health problems such as: heart attack, HBP, type 2 diabetes, and family history of chronic diseases. A higher healthy lifestyle index, indicating that the study participants have followed almost all of the studied recommendations related to the lifestyle, was a significant correlate of cholesterol level testing. Our study indicates the need to conduct campaigns to inform people about the effectiveness of lifestyle changes and about the necessity to control BP and cholesterol levels. Such actions should take into account the needs of the disadvantaged population and should especially target men, people with existing chronic diseases, and those with unfavorable lifestyle characteristics.

With respect to the socially-disadvantaged population, the social assistance institutions and outpatient clinics are the best places to conduct activities that promote healthy lifestyle. Studies suggest that the social community service organizations setting is a potentially effective way to reach disadvantaged people with an unfavorable lifestyle [74]. They have frequent and often continuous contact with their clients. Additionally, general practitioners should play the leading role in routine assessment of the patients’ risk (including lifestyle and health status assessment), recording it and referring to more specialized institutions if needed. Computerized systems, supported through government funding, should be considered in general practice and hospital settings. Health departments can also take a leading role in providing the social assistance staff with training in the delivery of a healthy lifestyle message at no cost to the service.

The most commonly used strategies to promote lifestyle changes can cover risk assessment, increasing awareness, emotional support and encouragement as well as referrals to specialists. Group counseling promoting a healthy lifestyle (focusing on a recreational physical activity, healthy/balanced diet, avoiding alcohol consumption, non-smoking, but also on promoting health examination/checkup, including weight control and BP and cholesterol testing) could be effective. Some general educational activities/campaigns dedicated to rural or disadvantaged populations are also crucial. There is evidence of an ‘inverse care law’ whereby health services are more accessible in the more affluent (and often less needy) areas and that patients from lower socioeconomic positions receive less preventive care from their doctors [74]. This underlined that such activities should be easily accessible, at low or no costs, especially in the case of people with a lower SES.

## Figures and Tables

**Table 1:** Characteristics of the study participants.

Variables	Total N = 1710 100%		Blood Pressure Testing Yes = 1114 65.1%		Cholesterol Level Testing Yes = 460 26.9%	
	N	%	N	%	N	%
Sex						
Male	568	33.2	343	60.4	131	23.1
Female	1142	66.8	771	67.5	329	28.8
Age (years)						
<30	194	11.3	116	59.8	27	13.9
30-39	725	42.4	462	63.7	165	22.8
40-49	578	33.8	385	66.6	192	33.2
50-59	213	12.5	151	70.9	76	35.7
Subjective health state						
Fair/rather fair	1121	65.5	685	61.1	252	22.5
Neither fair nor poor	407	23.8	300	73.7	135	33.2
Rather poor/poor	182	10.6	129	70.9	73	40.1
Number of health problems						
0	231	13.5	139	60.2	44	19.0
1-3	900	52.6	564	62.7	227	25.2
4-6	448	26.2	323	72.1	141	38.2
>7	97	5.7	66	68.0	41	42.3
Missing data	34	2.0	22	64.7	7	20.6
Heart attack						
Yes	22	1.3	18	81.8	15	68.2
No	1688	98.7	1096	64.9	445	26.4
High blood pressure						
Yes	197	11.5	172	87.3	105	53.3
No	1513	88.5	942	62.3	355	23.5
Total HLIDiabetes						
Yes	42	2.5	34	81.0	27	64.3
No	1668	97.5	1080	64.7	433	26.0
Family history of chronic diseases						
Yes	1175	68.7	803	68.3	346	29.4
No	318	18.6	187	58.8	58	18.2
I don’t know or missing	217	12.7	124	57.1	56	25.8
Smoking HLI						
1	1071	62.6	711	66.4	308	28.8
0	637	37.3	402	63.1	152	23.9
Missing data	2	0.1	1	50.0	0	0.0
Diet HLI						
1	160	9.4	106	66.3	53	33.1
0	1550	90.6	1008	65.0	407	26.3
Recreational physical activity HLI						
1	445	26.0	301	67.6	126	28.3
0	1238	72.4	798	64.5	326	26.3
Missing data	27	1.6	15	55.6	8	29.6
Alcohol HLI						
1	950	55.6	634	66.7	286	30.1
0	694	40.6	445	64.1	162	23.3
Missing data	66	3.8	35	53.0	12	18.2
BMI HLI						
1	732	42.8	491	67.1	201	27.5
0	978	57.2	623	63.7	259	26.5
Total HLI						
0	154	9.0	100	64.9	38	25.7
1	401	23.5	253	63.1	97	24.2
2	546	31.9	349	63.9	141	25.8
3	331	19.4	231	69.8	94	28.4
4	173	10.1	122	70.5	66	38.2
5	11	0.6	9	81.8	5	45.5
Missing data	94	5.5	50	53.2	19	20.2

HLI—healthy lifestyle indicator. BMI—body mass index.

**Table 2:** Chance of performing blood pressure and cholesterol level testing.

Variable	Chance of Performing Blood Pressure Testing				Chance of Performing Cholesterol Level Testing			
	Unadjusted Model		Adjusted Model		Unadjusted Model		Adjusted Model	
	OR (95% CI)	*p*-Value	OR (95% CI)	*p*-Value	OR (95% CI)	*p*-Value	OR (95% CI)	*p*-Value
Sex								
Male	1.00 Ref.		1.00 Ref		1.00 Ref.		1.00 Ref	
Female	1.36 (1.10-1.68)	0.004	1.51 (1.17-1.96)	0.002	1.34 (1.06-1.70)	0.01	1.36 (1.02-1.81)	0.04
Age (years)								
<30	1.00 Ref		1.00 Ref		1.00 Ref		1.00 Ref	
30-39	1.17 (0.85-1.63)	>0.05	1.02 (0.70-1.52)	>0.05	1.83 (1.17-2.84)	0.008	1.91 (1.13-3.25)	0.02
40-49	1.34 (0.96-1.87)	>0.05	0.97 (0.64-1.48)	>0.05	3.08 (1.98-4.79)	<0.001	2.64 (1.54-4.54)	<0.001
50-59	1.62 (1.07-2.43)	0.02	1.11 (0.65-1.88)	>0.05	3.46 (2.11-5.67)	<0.001	2.67 (1.44-4.95)	0.002
Subjective health state								
Fair/rather fair	1.00 Ref		1.00 Ref		1.00 Ref		1.00 Ref	
Neither fair nor poor	1.77 (1.38-2.28)	<0.001	1.54 (1.12-2.11)	0.008	1.71 (1.33-2.19)	<0.001	1.15 (0.83-1.58)	>0.05
Rather poor/poor	1.60 (1.13-2.26)	0.008	1.21 (0.76-1.91)	>0.05	2.30 (1.66-3.20)	<0.001	1.38 (0.89-2.14)	>0.05
Number of health problems								
0	1.00 Ref		1.00 Ref		1.00 Ref		1.00 Ref	
1-3	1.11 (0.82-1.49)	>0.05	1.01 (0.72-1.41)	>0.05	1.44 (1.00-2.06)	0.05	1.26 (0.83-1.90)	>0.05
4-6	1.71 (1.22-2.39)	0.002	1.16 (0.77-1.75)	>0.05	1.95 (1.33-2.87)	<0.001	1.12 (0.70-1.79)	>0.05
>7	1.39 (0.84-2.30)	>0.05	0.86 (0.46-1.59)	>0.05	3.11 (1.85-5.23)	<0.001	1.62 (0.86-3.00)	>0.05
Heart attack								
Yes	2.41 (0.81-7.16)	>0.05	1.38 (0.37-5.09)	>0.05	5.98 (2.42-14.77)	<0.001	2.98 (1.05-8.46)	0.04
No	1.00 Ref		1.00 Ref		1.00 Ref		1.00 Ref	
High blood pressure								
Yes	4.72 (2.99-7.46)	<0.001	3.92 (2.32-6.64)	<0.001	3.76 (2.77-5.10)	<0.001	2.32 (1.61-3.34)	<0.001
No	1.00 Ref		1.00 Ref		1.00 Ref		1.00 Ref	
Diabetes								
Yes	2.63 (1.16-5.97)	0.02	1.29 (0.46-3.59)	>0.05	5.13 (2.70-9.73)	<0.001	3.33 (1.49-7.47)	0.004
No	1.00 Ref		1.00 Ref		1.00 Ref		1.00 Ref	
Family history of chronic diseases								
Yes	1.51 (1.17-1.95)	0.002	1.32 (0.99-1.75)	0.06	1.87 (1.37-2.56)	<0.001	1.46 (1.03-2.07)	0.03
No	1.00 Ref		1.00 Ref		1.00 Ref		1.00 Ref	
Total HLI								
0-3	1.00 Ref		1.00 Ref		1.00 Ref		1.00 Ref	
4-5	1.31 (0.94-1.84)	>0.05	1.15 (0.79-1.68)	>0.05	1.80 (1.31-2.48)	<0.001	1.67 (1.16-2.41)	0.006
